# Attention in the temporal domain: a phase-coding mechanism controls the gain of sensory processing

**DOI:** 10.3389/fnhum.2013.00480

**Published:** 2013-08-19

**Authors:** Benjamin Morillon, Antoine Barbot

**Affiliations:** ^1^Department of Psychiatry, Columbia University Medical CenterNew York, NY, USA; ^2^Department of Psychology, New York UniversityNew York, NY, USA

## Introduction

We are constantly confronted with more information than our sensory systems, limited in resources, can effectively process. *Selective attention* is the means by which our brain prioritizes the processing of behaviorally relevant information. Recent work in the visual domain indicates that it relies on a large variety of physiological mechanisms. At the single cell level selective attention is reflected by increased firing rate (Spitzer et al., 1988) and/or enhanced synaptic efficacy (Briggs et al., 2013). At the population level, the synchrony, variability, correlation structure, pooling efficiency, and/or response gain of neural activity is modulated (for a comprehensive overview of these mechanisms see Serences, 2011).

The extent to which these mechanisms can operate independently from each other is not known. To disambiguate this important issue, researchers try to identify and study each possible parameter and dimension that might play a role in selective attention. For instance, attention either acts by modulating sensitivity to the *content* of a stimulus (feature-based attention), or to the *frame* in which it is embedded, through spatial and temporal attention (Nobre et al., 2012). Although feature-based and spatial attention have since long ago been a subject of intense investigation (Carrasco, 2011) temporal attention has historically received little emphasis, in spite of its ubiquitous presence. Two recent studies from Prof. Nobre's group at Oxford University, comprised of a behavioral experiment (Rohenkohl et al., 2012) and its EEG-recording counterpart (Cravo et al., 2013), provide new evidence that temporal attention (expectation) affects early stages of visual processing and uncover how these effects are implemented at the neural level.

## Paradigms of temporal attention

Temporal attention is classically studied using rhythmic streams of stimuli, given that the temporal structure of external events can *entrain* attentional focus. Its influence on reaction time is well-characterized and commonly linked to improved action preparation or execution (Nobre et al., 2012). That is, in a simple model *y* = a^*^x + *b*, where decision “*y*” depends on stimulus “*x*” and two independent variables, temporal attention is usually modeled as modulating the *bias* “*b*” while having no effect on the *gain* “*a*,” hence referred to as an anticipatory bias effect that has no influence at the perceptual level. These studies generally report reaction time differences, but often with ceiling effects on accuracy. Interpretations are thus limited as effects on reaction time might reflect early or late processing-stage modulations.

Rohenkohl et al. (2012) and Cravo et al. (2013) showed that temporal attention improves the quality of sensory information. They cleverly capitalized on the fact that only higher accuracy coupled with faster reaction time surely indicates enhanced sensory processing. To closely measure whether and how temporal attention modulates performance, they designed a visual detection task of briefly presented (50 ms) noise-embedded Gabor patches. They parametrically modulated the signal-to-noise contrast in order to characterize the full contrast sensitivity curve, from chance-level to asymptotic performance. Moreover, to characterize the influence of temporal attention, observers had to detect target stimuli embedded in rhythmic (2.5 Hz) or arrhythmic streams of Gaussian noise patches. This manipulation allowed observers to be able to expect (or not) the exact moment at which target stimuli could occur.

## Temporal attention increases contrast sensitivity

Behavioral results of Rohenkohl et al. (2012), replicated in Cravo et al. (2013), show that observers are more accurate during the rhythmic condition. Importantly, this increase in performance with temporal attention was associated with shorter reaction times, ruling out speed-accuracy trade-off effects (i.e., faster but worse performance), which correspond to a modulation of the response bias. Two complementary analyses further confirmed this result: first, capitalizing on the fact that different visual contrasts had been presented, they established the *sigmoidal* relation between physical stimulus contrast and accuracy in each condition (rhythmic/arrhythmic) and compared the resulting psychometric functions. Temporal attention improved contrast threshold, i.e., in the rhythmic condition less contrast is required to obtain 75% accuracy. Second, they implemented a diffusion model, which incorporates both accuracy and reaction time to describe how sensory evidence is accumulated. They found that temporal attention increases the normalized accumulation rate (gain), but does not decrease the decision criterion (bias). This set of results shows that temporal attention enhances visual sensitivity, i.e., the signal-to-noise *gain* of the sensory evidence upon which decisions are made.

That temporal attention improves contrast sensitivity suggests there is neural modulation at early stages of visual processing. This finding raises a set of intriguing questions. First, what neurophysiological mechanism(s) underlie the effects of temporal attention? Gain modulation could be accounted for by many of them, two of the most obvious being signal enhancement and noise reduction. Second, is temporal attention interacting with or conditional upon feature-based or spatial attention? In their paradigm, the location and features (e.g., orientation and spatial frequency) of the target were constant across trials, hence associated with strong expectations and possibly other attentional influences than temporal ones. It was recently proposed that temporal attention acts by boosting the effects of spatial attention (Nobre et al., 2012). This seems like a reasonable assumption, as the visual cortex is retinotopically organized, which would suggest a multiplicative interaction between the two. However, whereas this could be the case in vision, which is spatial in essence, this possibility seems unlikely in the tonotopically organized auditory system, which primarily codes information content (frequency). Accordingly, it has also been shown that pitch judgments were influenced by the timing properties of auditory sequences (Jones et al., 2002). As a result, temporal attention might mainly act by boosting the effects of feature-based attention in the auditory domain.

These studies call forth the interesting question of (1) the uniqueness of the underlying mechanism(s) of temporal attention and (2) its interaction with other fundamental dimensions (space, feature) in the brain. Finally, as pointed out by Rohenkohl et al. (2012), temporal attention could also be differentially implemented when induced by non-rhythmic but still expectable streams (e.g., syncopation in music). While rhythm is an obvious case of temporal expectation, it can conflate *local* neural entrainment at the presentation rate with *top-down* attention modulations. If the former proves to be the operating mode of temporal attention at the sensory level, pushed to its climax during presentation of rhythmic streams of stimuli, the mechanisms underlying the latter would in turn still have to be discovered.

## Neural mechanisms of temporal attention

The relation between neurophysiology and behavior has only recently been investigated in the context of temporal attention. Studies have focused on low-frequency activity and reaction time measures (Lakatos et al., 2008, 2009; Stefanics et al., 2010). Cravo et al. (2013) complemented these results by recording EEG activity during the above-mentioned task and by using a trial-by-trial GLM approach. They report a co-modulation between contrast gain and slow oscillatory activity, thereby establishing that a *phase-coding* mechanism is at the origin of the increased *quality* of sensory processing. The authors focused on pre-stimulus delta (1–4 Hz) activity, a band that encompasses the average stimulation rate (2.5 Hz). First, they found no pre-stimulus amplitude difference between rhythmic and arrhythmic conditions, which indicates that rhythmic entrainment at the stimulation rate does not translate into large-scale amplitude fluctuations, but probably operates via sub-threshold oscillations of sensory neurons (Lakatos et al., 2013).

Second, they extracted for both conditions the *contrast gain* (one minus the contrast threshold obtained from the behavioral sigmoid functions), and fitted it to their neural data. Interestingly contrast gain correlated with the delta *pre-stimulus* phase in the rhythmic, but not arrhythmic condition, indicating that the phase of delta waves predicts the quality of subsequent target processing, but only when temporal expectations are formed. Moreover, this correlation was maximal for the phase corresponding to an entrainment precisely matching the stimulation rate, ensuring an optimized excitability state at time of stimulus occurrence (Schroeder and Lakatos, 2009). Given these results, the authors concluded that phase entrainment of low-frequency oscillations to external sensory cues is the mechanism by which temporal attention increases contrast sensitivity.

Several mechanisms could account for the effect of temporal attention on contrast sensitivity. Here, Cravo et al. (2013) investigated the possible contribution of signal amplification and/or noise suppression. They reasoned that the parametric modulation of the signal-to-noise contrast used in their paradigm originated from the coupling of a constant noise with different signal intensities. Hence, a contrast-independent effect of temporal attention would suggest a noise suppression mechanism, whereas a contrast-dependent effect would favor signal amplification. By studying contrast-dependent EEG responses to the targets, the authors found suggestive evidence for the latter. Indeed, they found that the effect of temporal attention on visual responses at 200–300 ms following target onset grew linearly with contrast strength. In light of other studies, this suggests that temporal and spatial attention could operate via complementary mechanisms, respectively, through response enhancement and noise reduction (Cohen and Kohn, 2011; Wyart et al., 2012a), and thus be independent. However, as spatial attention was held constant in this study, it remains to be determined whether and how temporal and spatial attention interact, and if a single neurophysiological mechanism underlies both response enhancement and noise suppression.

## Future directions

These results support the idea that temporal attention fluctuates at the stimulation rate, optimizing the signal gain at moments of possible stimulus occurrence. This increased contrast sensitivity results in a better accumulation rate of sensory evidence, thereby leading to improved accuracy and faster reaction time. However, the authors leave open a fundamental question: is there an *explicit* neural substrate of temporal attention or is it only coded in the *carrier* (stimulation) frequency? Noteworthily, another study by the same group showed that alpha power follows the time course of temporal attention, as indexed by delta phase, by means of a phase-amplitude coupling dependency (Rohenkohl and Nobre, 2011). While phase-amplitude coupling is a likely substrate of the influence of temporal attention on information processing, the specific frequencies involved could in turn only reflect task structure. The carrier frequency follows the stimulation rate across a wide frequency range (at least from 1 to 12 Hz) (Lakatos et al., 2013). The kind of information (content vs. temporal) encoded in the stimulation rate also seems flexible (Stefanics et al., 2010). The case of the modulated frequency (the above-reported alpha) is less clear: it could depend on the modality involved (e.g., visual vs. auditory), the stage where temporal attention operates (e.g., primary vs. higher-order regions), or could instead be a general mechanism for target enhancement and/or distractor suppression (Haegens et al., 2011).

A next step would thus be to search for *hardwired* constraints governing attention mechanisms. Unlike sensory processing, sequential information integration at a central stage appears to be limited to around 2 Hz, as exemplified by psychological refractory phenomena such as the *attentional blink* (Wyart et al., 2012b). Would there be analogous constraints limiting temporal attention? This question might be more complex than it appears, as durations shorter and longer than 2 s are hypothesized to be encoded by different neural networks (Morillon et al., 2009). The constraints could thus depend on the neural system involved, itself being recruited according to the task parameters. We already mentioned the importance of testing the respective influences of content, spatial, and temporal dimensions in a single study, to confirm their complementary role on sensory processing, uncover the specific mechanisms that govern each of them, and quantify how they might interact (e.g., additive vs. multiplicative relation). Generally, the non-studied dimension(s) are neither controlled for nor randomized across trials, representing thus an important source of confounds. A first study in that direction recently showed that feature-based and spatial attention seem to affect the activity of visual local populations similarly (at least with regard to the firing rate and the inter-neuronal correlation structure) but differ in that spatial attention acts more locally (Cohen and Maunsell, 2011).

Maybe more important is the fact that studies on attention often conflate *behavioral relevance* and *signal probability*, attention being manipulated via the forthcoming stimulus' prior knowledge (Summerfield and Egner, 2009). That is, the build-up of a strong expectation (e.g., the rhythm) is used to boost the effects of attention. Although the role of attention in perception has been well-characterized, little is known about the mechanisms of *sensory predictions* (Summerfield and Egner, 2009; Arnal and Giraud, 2012). Both attention and prediction influence sensory representations, but they improve the quality of sensory processing in behaviorally dissociable ways (Wyart et al., 2012a). Moreover, they supposedly have independent neurophysiological substrates, modulating different processing stages along the hierarchy (Friston, 2009). As it is difficult to exclude one without affecting the other, a recently conducted approach was to control them in an orthogonal way (Kok et al., 2012; Wyart et al., 2012a). It was proposed that the influence of attention and prediction on perceptual sensitivity grows and shrinks with signal strength, respectively (Wyart et al., 2012a). This would provide a way to disambiguate between the two with a single parameter. However, for now the neural mechanisms underlying (time, space, and content) prediction remains to be established.
